# Multiple Aneurysms AnaTomy CHallenge 2018 (MATCH)—Phase Ib: Effect of morphology on hemodynamics

**DOI:** 10.1371/journal.pone.0216813

**Published:** 2019-05-17

**Authors:** Samuel Voß, Oliver Beuing, Gábor Janiga, Philipp Berg

**Affiliations:** 1 Department of Fluid Dynamics and Technical Flows, University of Magdeburg, Magdeburg, Germany; 2 Forschungscampus *STIMULATE*, Magdeburg, Germany; 3 Institute of Neuroradiology, University Hospital Magdeburg, Magdeburg, Germany; Technion Israel Institute of Technology, ISRAEL

## Abstract

**Background:**

Image-based blood flow simulations have been increasingly applied to investigate intracranial aneurysm (IA) hemodynamics. However, the acceptance among physicians remains limited due to the high variability in the underlying assumptions and quality of results.

**Methods:**

To evaluate the vessel segmentation as one of the most important sources of error, the international Multiple Aneurysms AnaTomy CHallenge 2018 (MATCH) was announced. 26 research groups from 13 different countries segmented three datasets, which contained five IAs in total. Based on these segmentations, 73 time-dependent blood flow simulations under consistent conditions were carried out. Afterwards, relevant flow and shear parameters (e.g., neck inflow rate, parent vessel flow rate, spatial mean velocity, and wall shear stress) were analyzed both qualitatively and quantitatively.

**Results:**

Regarding the entire vasculature, the variability of the segmented vessel radius is 0.13 mm, consistent and independent of the local vessel radius. However, the centerline velocity shows increased variability in more distal vessels. Focusing on the aneurysms, clear differences in morphological and hemodynamic parameters were observed. The quantification of the segmentation-induced variability showed approximately a 14% difference among the groups for the parent vessel flow rate. Regarding the mean aneurysmal velocity and the neck inflow rate, a variation of 30% and 46% was observed, respectively. Finally, time-averaged wall shear stresses varied between 28% and 51%, depending on the aneurysm in question.

**Conclusions:**

MATCH reveals the effect of state-of-the-art segmentation algorithms on subsequent hemodynamic simulations for IA research. The observed variations may lead to an inappropriate interpretation of the simulation results and thus, can lead to inappropriate conclusions by physicians. Therefore, accurate segmentation of the region of interest is necessary to obtain reliable and clinically helpful flow information.

## 1. Introduction

Intracranial aneurysms (IAs) became a popular research topic for biomedical engineers as computational fluid dynamics (CFD) can provide detailed hemodynamic information for a potential application in rupture risk assessment [1–5]. However, due to the lack of individual-patient information (e.g., missing flow or pressure waveforms), simulation boundary conditions were mostly based on non-physiological assumptions. As a consequence of this non-standardized situation, the literature is filled with a rapidly increasing number of related publications—something that may confuses physicians who can hardly identify the clinically relevant findings [6,7].

To evaluate the capabilities of image-based hemodynamic simulations and assess the variability of related research groups regarding their flow predictions, the concept of a challenge was introduced by Steinman et al. in 2012 [8]. In this challenge, 25 groups from all over the world participated. Numerical velocity and pressure results for a giant IA were gathered, and the comparison revealed that the predictions were consistent independent of the numerical solver that was used. However, further investigations were required to obtain wall shear stresses (WSS) and other clinically relevant hemodynamic parameters. Hence, a follow-up challenge (organized by Janiga and Berg) addressed this need and showed a good agreement among research groups regarding velocity and pressure. This agreement was able to confirm the usability of the underlying computational methodology when patient-specific geometries are provided [9,10]. Furthermore, normalized WSS qualitatively agreed among the groups as well.

To include the effect of vessel segmentation, Kono and Valen-Sendstad announced another international aneurysm challenge in 2015 where they provided only the raw DICOM datasets of five middle cerebral artery (MCA) aneurysms, and not the already segmented aneurysm surface as in the two previous challenges. They demonstrated, that segmentation quality and size of the considered domain varied substantially between groups [11]. To investigate the effect of the segmentation on hemodynamic simulations in more detail, the latest international competition (“Multiple Aneurysms AnaTomy CHallenge 2018—MATCH”, see S1 File) consisted of two phases. Twenty-six research groups took part in the first phase, which focused on the segmentation of five IAs in a single patient to maintain identical imaging conditions. As in the previous challenge, clear inter-groups differences were revealed. For example, only one group accurately reconstructed the neck of the ruptured aneurysm [12]. The second phase of MATCH focused on determining the rupture risk [13]. Since this is not relevant for the work presented here, it will not be discussed further below.

As MATCH revealed that segmentation quality has a major impact on subsequent hemodynamic simulations, a detailed investigation of this necessary processing step is required. This study is an extension of the first phase of MATCH with the aim to perform a standardized post processing for a better comparability of segmentation results from different groups. The segmentation results provided by the 26 participants serve as a basis to carry out blood flow simulations under identical conditions. Thus, the impact of vessel segmentation on hemodynamic simulations can be quantified.

## 2. Methods

### 2.1 Case description

All five IAs were detected in a female patient, who presented with subarachnoid hemorrhage. Four were located in the anterior and one in the vertebrobasilar circulation. Aneurysm A (5.6 mm) was located in the right MCA, while aneurysm B (1.5 mm) was located just proximal to it. On the left anterior side, aneurysm C and D were located in the MCA as well (4.4 mm; 4.6 mm). The fifth aneurysm, titled case E, appeared in the left posterior inferior cerebellar artery (4.9 mm). Aneurysm A and B were successfully treated by clipping, while coiling was carried out for aneurysms C, D, and E.

3D rotational angiography was carried out on an Artis Q angiography system (Siemens Healthineers AG, Forchheim, Germany) with 0.28 mm (iso) spatial resolution. Afterwards, the raw image data were reconstructed on a syngo X Workplace (Siemens Healthcare GmbH, Forchheim, Germany) using an ‘HU auto’ kernel [14]. This study is based on surface information previously derived from clinical image data. As data usage is retrospectively and permanently anonymized, the local institutional review board deemed the study exempt from the requirement for approval.

### 2.2 Segmentation

In total, 26 groups from 13 countries (see Table 1) submitted three segmentation results each. Due to distortion issues of the geometry, two segmentations by Group 3 could not be processed any further. Additionally, due to a limited vascular domain Group 5 had to be rejected as well. Thus, 73 (26 groups times three datasets minus five outliers) segmentations were included in this investigation (S1–S3 Dataset). For four segmentations (Group 7 and 22 regarding the left and Group 7 and 11 regarding the right anterior circulation) the internal carotid artery was extended to ensure equal inflow conditions. To obtain an objective comparison and to reduce bias in numerical methods [15–17], all post-segmentation steps were carried out by the challenge organizers. For further information regarding the challenge announcement, participating groups and segmentation details, the authors refer to the S1 Table as well as the associated initial study [12].

**Table 1:** Origins of the MATCH participants. Note that the numbering (alphabetical order) does not correspond to the numbers in the results section in order to keep the anonymity of each group.

#	Institution	Country	#	Institution	Country
1	Macquarie University	Australia	14	Tohoku University Graduate of Medicine	Japan
2	Toronto Western Hospital	Canada	15	Tokyo University	Japan
3	Universidad Mayor, Santiago de Chile	Chile	16	Saitama Medical University General Hospital	Japan
4	Charité Berlin	Germany	17	Simula Research Laboratory	Norway
5	Dornheim Medical Images	Germany	18	Tambov State Technical University	Russia
6	University Hospital Kiel	Germany	19	Universitat Rovira i Virgili	Spain
7	University of Magdeburg	Germany	20	George Mason University, Fairfax	USA
8	University of Magdeburg	Germany	21	Houston Methodist Research Institute	USA
9	University Hospital Regensburg	Germany	22	Mayo Clinic Rochester	USA
10	University of Hong Kong	Hongkong	23	Stanford University	USA
11	Budapest University	Hungary	24	Texas A&M University	USA
12	Medtronic Engineering Innovation Centre	India	25	University at Buffalo	USA
13	University of Parma	Italy	26	University of Texas at San Antonio	USA

### 2.3 Spatial discretization

All segmentation results were spatially discretized using identical settings. Volumetric meshing was carried out with STAR CCM+ 12.02 (Siemens PLM Software Inc., Plano, TX, USA), while unstructured grids based on polyhedral and prism cells were generated (base size Δx = 0.07–0.09 mm). Particularly, the vessel walls were resolved appropriately to account for the steep velocity gradients [18]. On average, this resulted in meshes with 2.8 (left anterior circulation), 1.9 (posterior circulation), and 2.6 (right anterior circulation) million cells depending on the individual discretized volume.

### 2.4 Hemodynamic simulations

Based on the spatial discretization of each dataset, hemodynamic simulations were carried out using the finite volume flow solver STAR CCM+ 12.02. At each inlet, a time-varying flow rate from Cebral et al. [19] was applied. Note that each inlet was identically extruded in the normal direction by at least 10 times the nominal inlet diameter leading to a developed flow profile. Thus, it justifies the use of a plug profile as demonstrated in Berg et al. [10]. To account for the different inlet cross-sections resulting from the group-dependent segmentation results, the flow rate was adjusted according to Valen-Sendstad et al. [20], see S2 Table for the corresponding waveforms of each group. A constant time-step of Δt = 1 ms was used in each simulation.

As it is not possible to precisely measure IA or arterial wall thicknesses and wall motion in-vivo even with present state-of-the-art techniques, a non-flexible behavior was assumed [21,22]. It must be noted that the number of outlets ranged from three to seven and four to eight for the right and left anterior circulation, and two to three for the posterior circulation. In order to avoid an overestimation of the effect due to a varying number of outlets the decision was made to apply the most common approach regarding outlet boundary conditions, and thus a zero-pressure condition was defined.

Flow was assumed to be laminar, and blood was treated as an incompressible (*ρ* = 1055 kg/m^3^), Newtonian (*μ* = 0.004 Pa s) fluid. For each of the 73 time-dependent simulations, three cardiac cycles were calculated with only the last cycle being used in the analysis.

### 2.5 Analysis

To assess the variability of the hemodynamic results, both qualitative and quantitative comparisons were carried out for flow and shear related parameters.

First, the vessel centerlines (extracted using the vascular modeling toolkit [23]) were used to quantify the global inter-group variability. In this regard, the local maximum inscribed sphere radius (segmentation) and the local velocity magnitude (hemodynamics) were evaluated and the standard deviations were calculated.

Second, morphological (ostium area, parent vessel area proximal to ostium, aneurysm volume, non-sphericity index), and cycle-averaged hemodynamic (aneurysm neck inflow rate, parent vessel flow rate, spatial mean aneurysm velocity) parameters were quantified [24]. Here, the ostium is defined as the smallest common plane between the aneurysms and the corresponding parent vessels in order to make the results comparable.

Third, the iso-surface velocities, time-averaged wall shear stresses (AWSS), and the oscillatory shear index (OSI) were presented to observe the individual flow and shear structures. Finally, boxplots of relevant morphological and hemodynamic parameters reveal the variability of simulation results induced by segmentation differences. See Eqs 1–3 for the definition of the morphologic and hemodynamic quantities:

- Equation 1: Non-sphericity index (NSI)
  $$\text{NSI}=1-{\left(18\pi \right)}^{\frac{1}{3}}\;V^{\frac{2}{3}}/S$$ (1)
  with the volume V and the surface area S of the corresponding aneurysm.
- Equation 2: Time-averaged wall shear stresses (AWSS)
  $$AWSS=\frac{1}{T}\int_{0}^{T}|WSS|dt$$ (2)
  with the period time of one cardiac cycle T and the instantaneous wall shear stress vector WSS.
- Equation 3: Oscillatory shear index (OSI)
  $$OSI=\frac{1}{2}\left\{1-\frac{\frac{1}{T}|\int_{0}^{T}WSS\;dt|}{\frac{1}{T}\int_{0}^{T}|WSS|dt}\right\}$$ (3)

## 3. Results

### 3.1 Centerline based analysis of segmentation and hemodynamics

**Fig 1:**
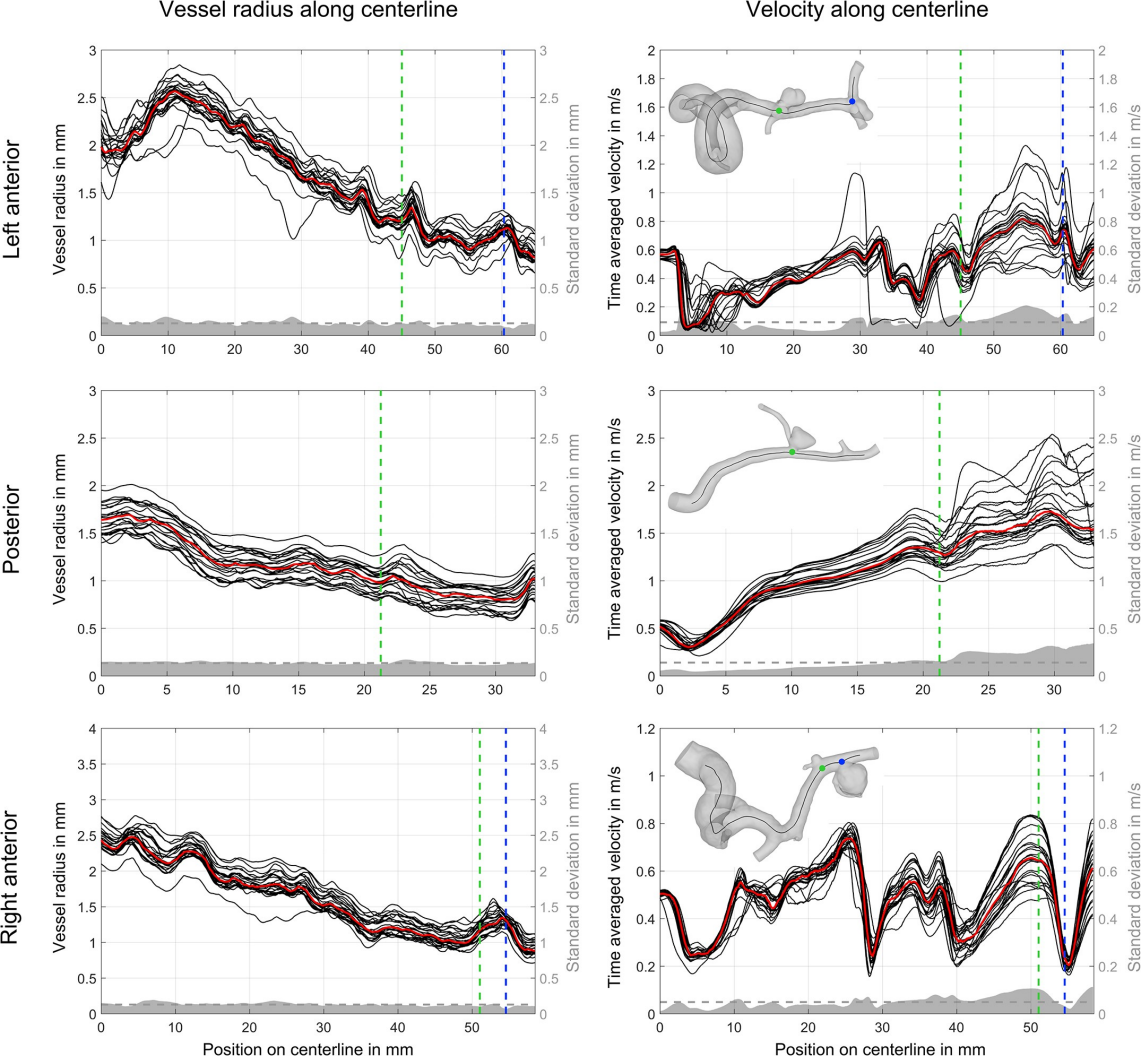
Comparison of vessel radius and velocity magnitude values for each group along the centerlines of the aneurysm carrying parent vessels in the anterior and posterior circulation. Median radius and velocity is shown as a red line. The vertical dashed lines indicate the location of the corresponding aneurysm (aneurysm A and B in the right anterior, aneurysm C and D in the left anterior, and aneurysm E in the posterior circulation). Furthermore, the standard deviation is presented in gray with the mean value using a horizontal dashed line.

First, the local maximum inscribed sphere radius was calculated along each centerline (see Fig 1, left). Proximal vessels (e.g., left and right internal carotid artery, vertebral artery) have larger radii than distal ones. A narrow band of radii curves is observed in all but a few outliers. The median radius curve is shown in red. The mean standard variation (gray dashed lines) is approx. 0.13 mm (left anterior: 0.126 mm, posterior: 0.133 mm and right anterior: 0.126 mm). This is consistent for all three datasets, independent of the local radii.

Second, velocity magnitudes calculated along the centerlines were compared. As presented in Fig 1, right, a good agreement among the groups is visible in the proximal regions of the investigated vascular domains. At the inlet, the velocity between groups is identical due to the inlet treatment. However, as the centerline length increases, the velocity variability also increases. This is particularly true in the area of and distal to the aneurysms. Standard deviations are always higher here compared to the corresponding mean values. In contrast to the local vessel radii, the standard deviation is less consistent along the centerline.

### 3.2 Aneurysm specific analysis of segmentation and hemodynamic

**Fig 2:**
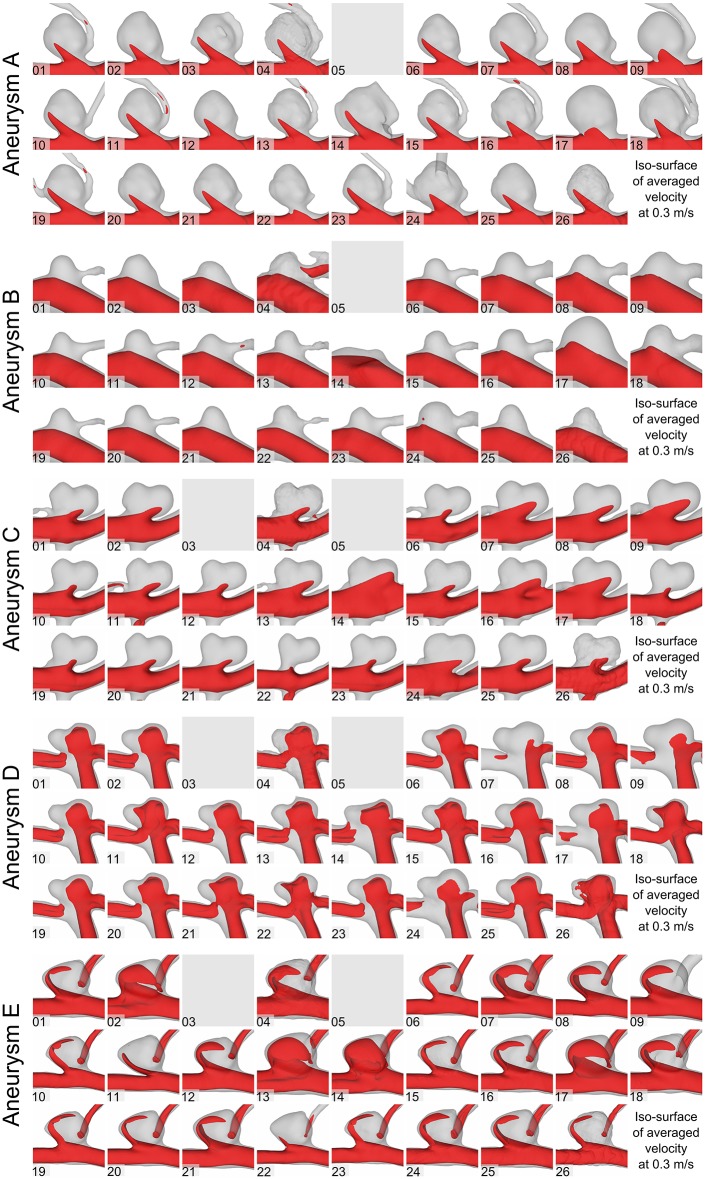
Assessment of the qualitative flow variability based on time-dependent hemodynamic simulations. Iso-surface velocities using a velocity threshold of 0.3 m/s for aneurysms A-E.

Based on the 73 time-dependent blood flow simulations, clear differences in the flow structures were observed. Fig 2 illustrates the time-averaged velocity based on iso-surfaces of each group for aneurysms A-E. As expected, relatively narrow aneurysm necks lead to a faster inflow jet for the bifurcation aneurysm (e.g., aneurysm D, Group 22), whereas wider necks decrease the corresponding inflow jet (e.g., Group 7). As the inflow boundary condition is scaled to the vessel diameter, these differences result from the segmentation alone. Furthermore, artifacts in the segmentations (such as the melted surfaces of the aneurysm dome and the parent vessel) result in unphysiological flow behavior (Fig 2, Group 4).

**Fig 3:**
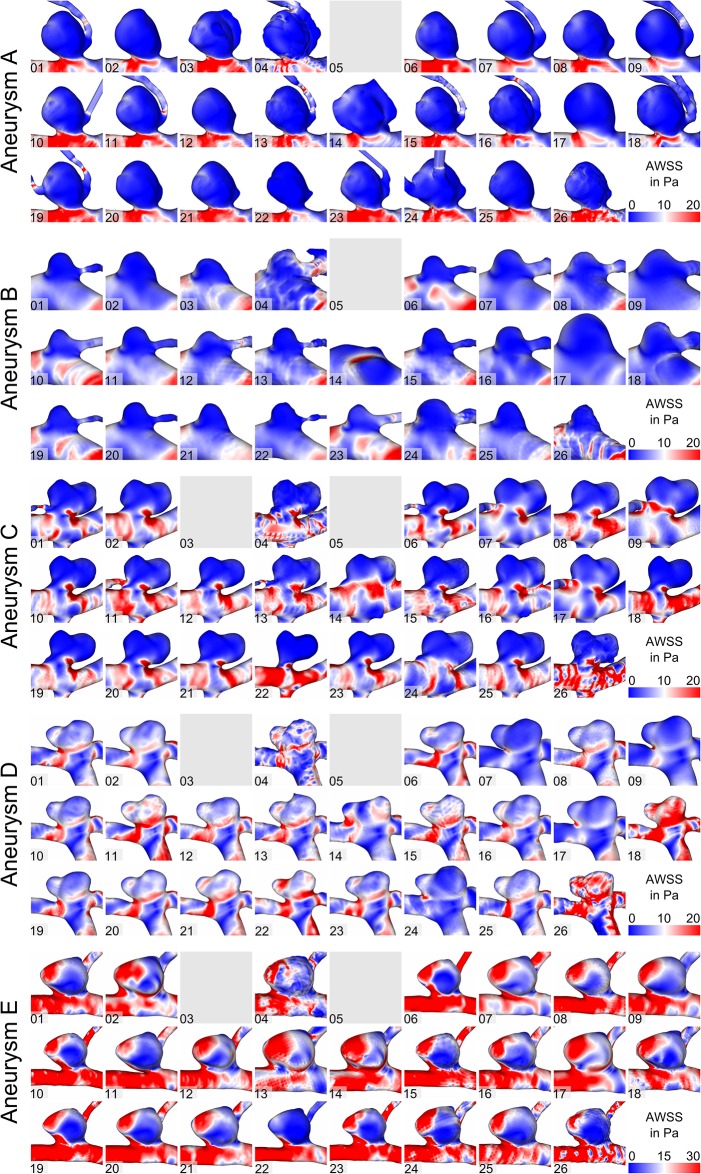
Assessment of the time-averaged wall shear stresses for aneurysms A-E.

**Fig 4:**
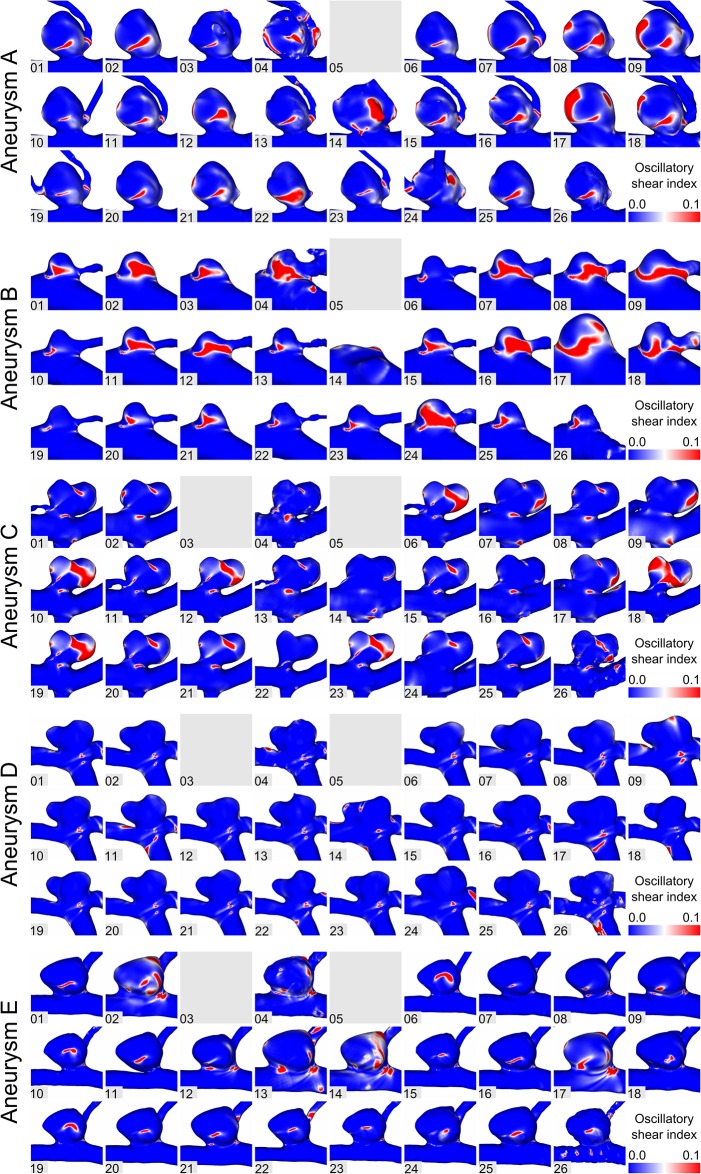
Assessment of the oscillatory shear index for aneurysms A-E.

In addition to the flow differences, the effect of segmentation on WSS was assessed. As shown in Fig 3, time-averaged WSS patterns are displayed, which can be seen for all aneurysms. Similar to previous observations, vessel surface reconstructions highly influence the subsequent hemodynamic predictions. While some segmentations experience only slightly increased WSS in the area of the aneurysm necks (e.g., aneurysm C; Groups 6, 18, 22), others show high values in the dome region as well (Group 14, 24). Furthermore, variability in the oscillating shear stress is illustrated in Fig 4 for all aneurysms. This indicator for aneurysm rupture clearly varies depending on the underlying segmentation result. While large areas of increased OSI are visible in some groups (e.g., aneurysm C; Groups 10, 18, 19), blood flow simulations using the surfaces of other groups would result in almost no OSI enhancement (e.g., Groups 14, 16, 22).

**Fig 5:**
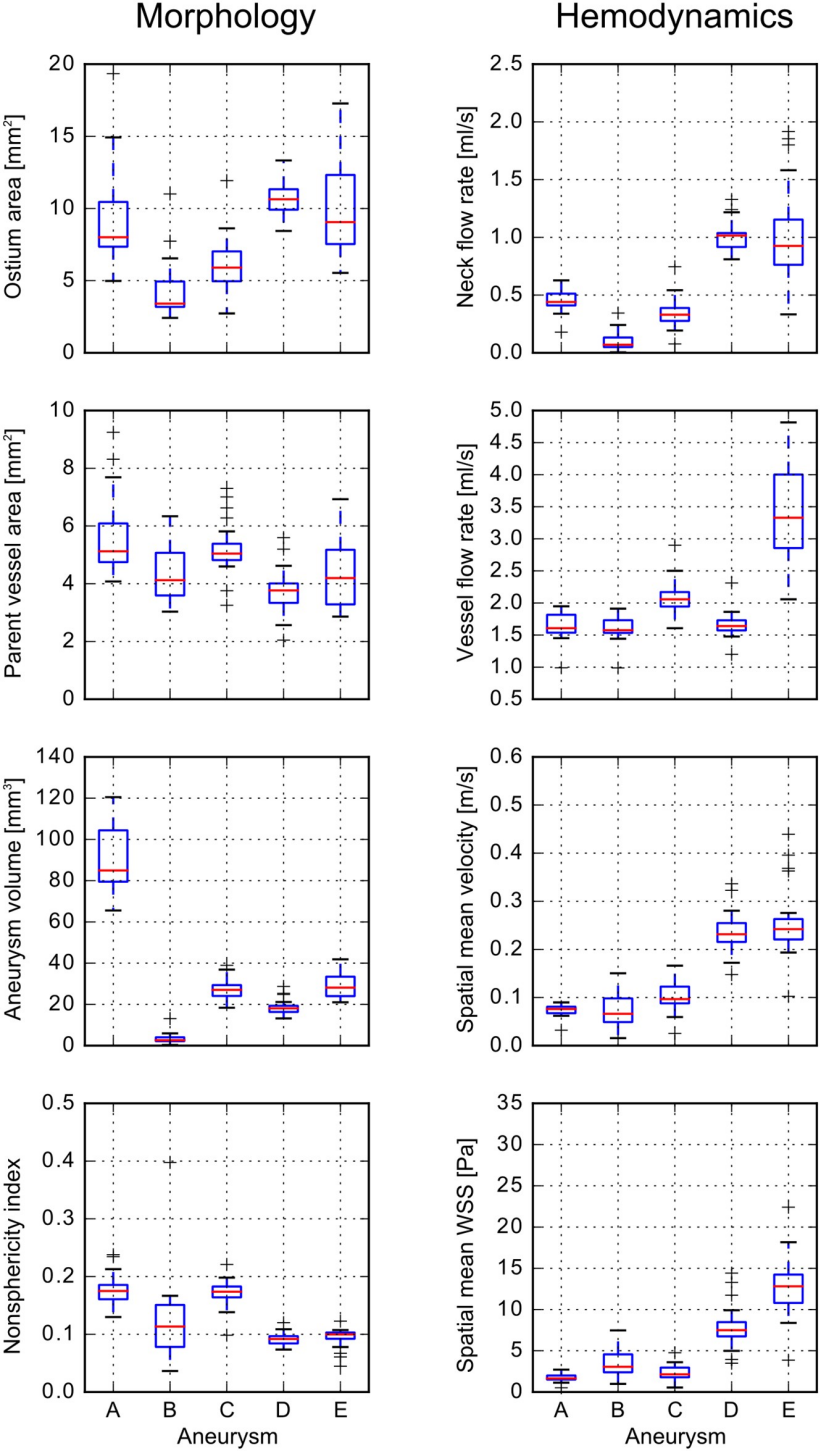
Boxplots of morphological and hemodynamic parameters. The morphological parameters (left) comprise of ostium area, parent vessel area, aneurysm volume, and non-sphericity index. Hemodynamic parameters (right) include the neck inflow rate, the parent vessel flow rate, the spatial mean velocity in the aneurysm, and the spatially and temporal averaged wall shear stress.

This qualitative impression is confirmed by the subsequent quantitative analysis. Fig 5 and Table 2 present four morphological and four hemodynamic parameters, respectively. In general, greater variability in morphology leads to an increased variability in hemodynamics, e.g., the ostium area affects the neck inflow rate. The lowest variability averaged over all aneurysms was found for the flow rate in the parent vessel (13.8%), followed by the parent vessel cross-sectional area (22.2%) and the non-sphericity index (23.8%). The aneurysm neck flow rate showed the highest variability (46.2%). Aneurysm A is the largest aneurysm by volume. Its variability is below the mean value in six out of eight parameters. Its ostium area varies by 40.3% between the groups. The segmentation of the smallest aneurysm B is very diverse, from very large (see Fig 2, group 17) to non-existent (group 14). Consequently, (except for parent vessel related parameters), the variability of aneurysm B is the highest with up to 113.8% regarding the aneurysm neck flow rate and 90% regarding the aneurysm volume. Aneurysms C to E are of similar size and relative standard deviations are primarily between 10–30%. Variability approaches 40% only with respect to aneurysm neck flow rate (C and E) and aneurysm spatial mean WSS (C).

**Table 2:** Morphological and hemodynamic parameters. Group’s median values for ostium area, parent vessel area (proximal to the aneurysm), aneurysm volume, and non-sphericity index are calculated, as well as the corresponding standard deviations. In addition, cycle-averaged aneurysm neck inflow rate, parent vessel flow rate (proximal to the aneurysm), spatial mean aneurysmal velocity, and spatial mean WSS are listed.

	Morphology		Hemodynamics	
	**Ostium area**		**Aneurysm neck flow rate**	
**Aneurysm**	Median [mm^2^]	rel. std.	Median [ml/s]	rel. std.
**A**	7.98	40.3%	0.44	21.5%
**B**	3.39	56.1%	0.07	113.8%
**C**	5.76	31.1%	0.33	40.5%
**D**	10.45	12.4%	1.02	12.7%
**E**	10.97	20.5%	0.93	42.3%
**mean**	**6.90**	**32.1%**	**0.56**	**46.2%**
	**Parent vessel cross-sectional area**		**Parent vessel flow rate**	
**Aneurysm**	Median [mm^2^]	rel. std.	Median [ml/s]	rel. std.
**A**	5.12	25.6%	1.61	12.4%
**B**	4.12	22.8%	1.58	11.4%
**C**	5.04	17.6%	2.06	12.7%
**D**	3.77	19.5%	1.64	11.9%
**E**	4.20	25.7%	3.33	20.8%
**mean**	**4.45**	**22.2%**	**2.04**	**13.8%**
	**Aneurysm volume**		**Aneurysm spatial mean velocity**	
**Aneurysm**	Median [mm^3^]	rel. std.	Median [m/s]	rel. std.
**A**	84.63	18.0%	0.08	14.8%
**B**	2.62	90.0%	0.07	52.8%
**C**	27.03	16.6%	0.10	32.5%
**D**	17.72	19.4%	0.23	18.0%
**E**	28.55	18.6%	0.24	29.0%
**mean**	**32.11**	**32.5%**	**0.14**	**29.4%**
	**Non-sphericity index**		**Aneurysm spatial mean WSS**	
**Aneurysm**	Median [–]	rel. std.	Median [Pa]	rel. std.
**A**	0.17	15.2%	1.64	27.7%
**B**	0.11	62.1%	3.06	51.3%
**C**	0.17	13.3%	2.14	42.9%
**D**	0.09	11.9%	7.50	33.5%
**E**	0.10	16.3%	12.82	27.8%
**mean**	**0.13**	**23.8%**	**5.43**	**36.6%**

## 4. Discussion

Growing computational resources allowed for an increased application of numerical methods to investigate neurovascular diseases. In particular, image-based hemodynamic simulations in cerebral aneurysms were carried out to either estimate the individual rupture probability or to assist during therapy planning. However, due to numerous simplifications during the modeling process, trust in the gained results remains to be limited among physicians.

To improve this situation, Jiang and Strother [25] assessed the influence of varying heart rates on IA hemodynamics. They demonstrated that local changes in the flow may be the consequence of different heart rates, but also of the aneurysm geometry. In a follow-up study, Valen-Sendstad et al. [20] evaluated the effect of inflow waveform scaling on subsequent computational fluid dynamics (CFD) simulations in IAs. Based on 37 internal carotid artery aneurysms, they found that a square law was the most consistent with physiological flow rates.

The assumption of a rigid vessel wall was discarded by Valencia et al. [26], Torii et al. [27], and Tezduyar et al. [28]. They performed fluid-structure-interaction (FSI) simulations in IAs, and observed significant differences in the wall stress distributions compared to the assumption of non-flexible walls. Additionally, Voß et al. [29] compared constant versus patient-specific cerebral wall thicknesses using FSI simulations, and demonstrated that increased wall stresses occurring at the aneurysm rupture site can only be revealed if realistic modeling is applied.

Regarding outflow settings, Chnafa et al. [30] recently investigated the effect of different outlet boundary condition concepts based on 70 middle cerebral artery aneurysms. Their comparison between the commonly used zero pressure assumption, Murray’s law (principle of minimum work), and an in-house model revealed clear differences. Thus, they concluded that the zero-pressure outlet method should be avoided and the integration of measured flow information (if available) is desired [31].

To further compare simulation settings in a more structured way and assess state-of-the-art capabilities of numerical methods, blinded challenges were frequently organized. These challenges focused on specific, clinically relevant questions. While the usability of existing flow solvers was extensively demonstrated [8,10], other aspects that had primary effects on the simulation quality were overlooked. In this regard, MATCH was announced to compare existing segmentation strategies for IAs, and evaluate their impact on subsequent blood flow simulations.

### Vascular flow variability

Segmentation and hemodynamic variability with respect to the vascular domains are quantified based on local vessel radii and velocity magnitudes along the centerline. While the vessel radii indicate a consistent relative standard deviation over the entire centerline of all three data sets, the variability of the velocity magnitude increased with centerline length.

Thus, different flow characteristics cannot be explained by local radius differences alone. Rather, the inconsistent consideration of side branches/bifurcations in the segmentations leads to increased variability in more distal locations (see also Berg et al. [12]).

### Aneurysmal flow behavior

Clear differences regarding the aneurysm-specific flow structures were observed within the 73 segmentations. In several groups, an overestimation of the aneurysm necks led to higher inflow rates, while small ostia led to concentrated inflow jets. Quantitatively, the segmentation-induced variability showed approximately a 14% difference among the groups for the parent vessel flow rate. Regarding the mean aneurysmal velocity and the neck inflow rate, a variation of 30% and 46% was observed, respectively. Additionally, AWSS was affected with rates varying between 28% and 51%, depending on the aneurysm in question.

Furthermore, a lacking segmentation of small side branches affected the intra-aneurysmal flow behavior, particularly in the vicinity of an IA. This was especially prominent for the largest aneurysm A, and the smallest aneurysm B. Regarding the type of aneurysm (lateral versus terminal), no relation to the grade of variability was found.

### Recommendations

Overall, this shows that inaccurate segmentation can either lead to flow results that are subject to uncertainties or even show wrong flow patterns due to segmentation artefacts. Thus, the following recommendations can be formulated for biomedical researchers investigating the individual flow conditions in IAs: 1) The importance of high-quality segmentation results cannot be emphasized enough when accurate hemodynamic predictions are desired. Specifically, the consideration of adjacent side branches and an appropriate reconstruction of the aneurysm neck, as well as morphological features such as blebs or daughter aneurysms, is crucial. Only then can large-scale studies containing high numbers of cases advance the knowledge of neurovascular diseases [32,33]. 2) During the analysis of clinically relevant morphological and hemodynamic parameters, it was found that these parameters are highly sensitive to the choice of the aneurysm ostium and neck curve, respectively. Therefore, a realistic separation of aneurysm and parent vessel is recommended when quantification of shape and flow parameters is carried out, e.g., using objective algorithms [34–36]. This is particularly required to avoid uncertainties due to subjective analysis.

If these recommendations regarding image-based segmentation and analysis are considered, the prediction error due to incautious and careless modeling can be reduced. Furthermore, it is recommended to formulate segmentation guidelines that must be respected in related studies in the future. In this regard, comparisons to a reliable ground truth solution are desired.

### Study limitations

This study has various limitations. First, patient-specific boundary conditions are required to perform realistic hemodynamic simulations. However, only the geometry of the cerebral vasculature was available with no information regarding flow waveforms, e. g., measured in the internal carotid arteries. Nevertheless, the aim of the study was the assessment of the hemodynamic variability due to segmentation differences. Thus, the applied setup assumed equal conditions in all 73 simulations.

Second, blood was treated as a continuous, Newtonian fluid with laminar flow conditions. Although some studies concluded that non-Newtonian behavior can affect the numerical results [37], others claim that there is no significant impact of available models [38]. Nevertheless, a consensus exists that compared to the influence of segmentation, the choice of blood treatment has only secondary effects.

Finally, all quantitative analyses contain the results of every group (excluding the one which was rejected for methodological reasons), and no outliers were excluded. Due to this, the variability of the investigated parameters represents the maximum differences, and an exclusion of clearly unrealistic solutions would decrease the actual error range.

## 5. Conclusions

The aneurysm challenge ‘MATCH 2018’ emphasizes the variability of existing segmentation approaches and its influence on subsequent hemodynamic simulations. Accordingly, it must be assumed that many of the previous studies can only be compared to a limited extent. In particular, it is shown how the wrong representation of key aneurysm surface features (e.g., neck or daughter aneurysms), or remaining imaging artefacts due to insufficient processing of the initial segmentation can lead to inaccurate qualitative and quantitative flow results. Therefore, the clinical applicability of image-based simulations may only be feasible when error-reduced, individualized blood flow predictions resulting from a consensus regarding an appropriate segmentation environment exists.

## Supporting information

S1 File Announcement of the Multiple Aneurysms AnaTomy CHallenge 2018 (MATCH). (PDF)

S1 Dataset Segmentations of the left anterior circulation. Surface meshes contributed by participants of MATCH Phase I.
(ZIP)

S2 Dataset Segmentations of the posterior circulation. Surface meshes contributed by participants of MATCH Phase I.
(ZIP)

S3 Dataset Segmentations of the right anterior circulation. Surface meshes contributed by participants of MATCH Phase I.
(ZIP)

S1 Table List of segmentation software and methods used by participants of MATCH Phase I. (XLSX)

S2 Table Inflow curves for the individual inlet cross-sections. (XLSX)
