# Supplementary material for: Multiple Aneurysms AnaTomy CHallenge 2018 (MATCH)—Phase Ib: Effect of morphology on hemodynamics
Source: PLoS One. 2019 May 17;14(5):e0216813. doi: 10.1371/journal.pone.0216813 (PMC6524809; doi:10.1371/journal.pone.0216813)
Supplement: S1 File — (PDF) [file pone.0216813.s001.pdf]

ICS 2018

June 06-08, 2018
Magdeburg, Germany

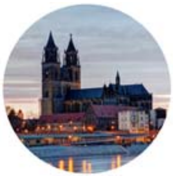

The 15th Interdisciplinary Cerebrovascular Symposium presents the

Multiple Aneurysms AnaTomy Challenge 2018 (MATCH)

Dear participants,

It is our great pleasure to announce the **Multiple Aneurysms AnaTomy Challenge 2018,** which aims to **MATCH** morphologic and hemodynamic analyses by aneurysm research groups from all around the world.

After a series of interesting and instructive competitions focusing on computational variabilities [1,2], aneurysm rupture risk assessment [3,4], and treatment outcome evaluation [5,6], this year's challenge focuses on the pre-simulative effect of segmentation approaches. Since the crucial effect of geometry on the subsequent blood flow simulations is well known, we would like to compare and quantify this influence with respect to different segmentation methodologies. Specifically, this challenge considers one patient with SAH and 5 intracranial aneurysms (see Figure 1). The ruptured aneurysm is known.

In the first phase of the challenge, participants are requested to apply their **segmentation** methods to the three provided 3D RA data sets (two anterior, one posterior) and to prepare the corresponding 3D segmented surfaces (please retain the original coordinate system and use the STL format). The organizers will use these segmentations to carry out hemodynamic simulations under identical conditions. This allows for the evaluation of geometric differences on the subsequent blood flow results.

In a second phase, participants are asked to perform **hemodynamic simulations** within their own geometries using arbitrary boundary conditions. Afterwards, the ruptured aneurysm shall be identified applying any desired criteria (e.g., hemodynamic parameters). Participation in Phase II is not mandatory.

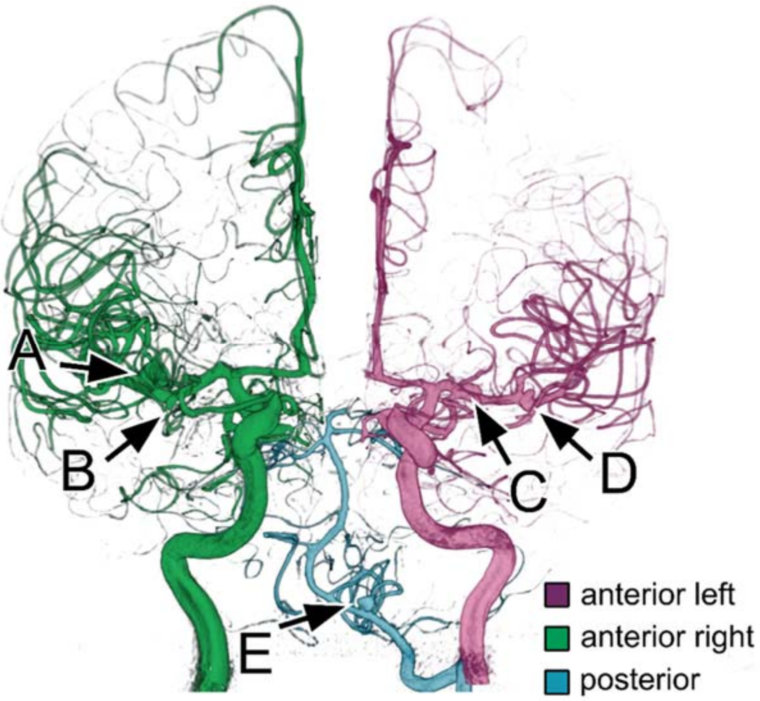

Figure 1: Illustration of the provided **MATCH** case containing five intracranial aneurysms. Arrows indicate the corresponding locations, denominated A to E.

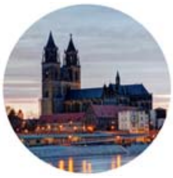

Challenge details

- **Data acquisition:** The three 3D RAs data sets containing five aneurysms can be requested with a short message to ics2018match@ovgu.de.
- **Phase I:** 3D segmentations of the aneurysm data sets using the *original coordinate system* are requested. Please submit the segmentation results of the aneurysms and adjacent vasculature in STL format. A consideration of at least 15 nominal vessel diameters proximal and 10 diameters distal to each aneurysm is required. Please further submit an informal abstract (max. 1 page) containing the author names, your affiliations and segmentation details. The following information is mandatory:
  1) Segmentation method, 2) software used, and 3) processing time from data import to final segmentation. Optional: parameters chosen depending on the segmentation method. No flow simulation is needed in this phase.
- **Phase II:** Hemodynamic simulations based on the participants' own segmentations and identification of the ruptured aneurysm using the criteria of your choice (e.g, hemodynamic parameters) are requested. Please submit a second informal abstract (max. 1 page) containing the authors' names, your affiliations, and simulation details. The following information is mandatory:
  1) Mesh resolution, 2) solver, 3) time-step size (if unsteady), 4) type of in- and outflow boundary condition, 5) viscosity/density, and 6) reasons for choosing a particular aneurysm as being the ruptured one (aneurysm A-E). Further details are optional.
- **Data submission:** Final segmentations as well as the corresponding abstracts should be sent to ics2018match@ovgu.de by the respective deadlines for each phase.

Important dates

- Announcement of the MATCH2018 — Fri, November 03, 2017
- Deadline for the submission of Phase I results — Mon, **January 08, 2018**
- Deadline for the submission of Phase II results — Fri, **February 2, 2018**
- Presentation of the results at ICS2018 — Thu, June 7, 2018

Depending on the resonance and the obtained results, our intention is to publish the outcome of **MATCH** in an international peer-reviewed journal, while for each participating group the corresponding authors will be considered.

We are hoping for numerous participants and are looking forward to your contributions.

Gábor Janiga (PhD), ICS18 President
Oliver Beuing (MD), ICS18 Co-President
Philipp Berg (PhD), ICS18 Chair of the Scientific Committee

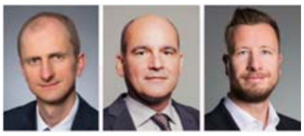

References

[1] Steinman DA, Hoi Y, Fahy P, Morris L, Walsh MT, et al.: Variability of computational fluid dynamics solutions for pressure and flow in a giant aneurysm: the ASME 2012 Summer Bioengineering Conference CFD Challenge. J Biomech Eng; 135(2):021016, 2013

[2] Berg P, Roloff C, Beuing O, Voss S, Sugiyama S, et al.: The Computational Fluid Dynamics Rupture Challenge 2013 - Phase II: Variability of Hemodynamic Simulations in Two Intracranial Aneurysms. J Biomech Eng; 137(12):121008, 2015

[3] Janiga G, Berg P, Sugiyama S, Kono K, Steinman DA: The Computational Fluid Dynamics Rupture Challenge 2013 - Phase I: prediction of rupture status in intracranial aneurysms. AJNR Am J Neuroradiol; 36(3):530-6, 2015

[4] Kono K, Valen-Sendstad K: Int. Aneurysm CFD Challenge 2015, http://cfdchallenge2015.c.ooco.jp/index2.html

[5] Cito S, Geers AJ, Arroyo MP, Palero VR, Pallarés J. et al.: Accuracy and reproducibility of patient-specific hemodynamic models of stented intracranial aneurysms: Report on the Virtual Intracranial Stenting Challenge 2011. Ann Biomed Eng. 43(1),154-167, 2015

[6] Kono K, Shojima M, Ohta M, Sakai N: Flow Diverter CFD Challenge 2016, http://cfdchallenge2015.c.ooco.jp
